# Passive Antibody Administration (Immediate Immunity) as a Specific Defense Against Biological Weapons

**DOI:** 10.3201/eid0808.010516

**Published:** 2002-08

**Authors:** Arturo Casadevall

**Affiliations:** * Albert Einstein College of Medicine, Bronx, New York, USA

**Keywords:** anthrax, antibody, biological weapons, bioterrorism, vaccines, immunization, immune, botulinum, toxin, brucellosis, *Brucella*, *Bacillus anthracis*, plague, *Yersinia*, smallpox, variola, tularemia, *Francisella tularensis*, ricin, Q fever, *Coxiella burnetii*

## Abstract

The potential threat of biological warfare with a specific agent is proportional to the susceptibility of the population to that agent. Preventing disease after exposure to a biological agent is partially a function of the immunity of the exposed individual. The only available countermeasure that can provide immediate immunity against a biological agent is passive antibody. Unlike vaccines, which require time to induce protective immunity and depend on the host’s ability to mount an immune response, passive antibody can theoretically confer protection regardless of the immune status of the host. Passive antibody therapy has substantial advantages over antimicrobial agents and other measures for postexposure prophylaxis, including low toxicity and high specific activity. Specific antibodies are active against the major agents of bioterrorism, including anthrax, smallpox, botulinum toxin, tularemia, and plague. This article proposes a biological defense initiative based on developing, producing, and stockpiling specific antibody reagents that can be used to protect the population against biological warfare threats.

Defense strategies against biological weapons include such measures as enhanced epidemiologic surveillance, vaccination, and use of antimicrobial agents, with the important caveat that the final line of defense is the immune system of the exposed individual. The potential threat of biological warfare and bioterrorism is inversely proportional to the number of immune persons in the targeted population. Thus, biological agents are potential weapons only against populations with a substantial proportion of susceptible persons. For example, smallpox virus would not be considered a useful biological weapon against a population universally immunized with vaccinia.

Vaccination can reduce the susceptibility of a population against specific threats provided that a safe vaccine exists that can induce a protective response. Unfortunately, inducing a protective response by vaccination may take longer than the time between exposure and onset of disease. Moreover, many vaccines require multiple doses to achieve a protective immune response, which would limit their usefulness in an emergency vaccination program to provide rapid prophylaxis after an attack. In fact, not all vaccine recipients mount a protective response, even after receiving the recommended immunization schedule. Persons with impaired immunity are often unable to generate effective response to vaccination, and certain vaccines may be contraindicated for them (1). For example, the vaccine against hepatitis B does not elicit an antibody response in approximately 10% of vaccines, and the percentage of nonresponders is substantially higher in immunocompromised persons (1).

Drugs can provide protection when administered after exposure to certain agents, but none are available against many potential agents of biological warfare. Currently, no small-molecule drugs are available that prevent disease following exposure to preformed toxins. The only currently available intervention that could provide a state of immediate immunity is passive immunization with protective antibody. Passive antibody therapy was widely used in the pre-antibiotic era but was largely abandoned with the advent of antimicrobial chemotherapy (2,3). In recent years, there has been a renaissance in the use of antibodies for therapy: 10 monoclonal antibodies (MAbs) are currently licensed and dozens are in the developmental pipeline (4). This article reviews the activity of humoral immunity against several biological agents, discusses the advantages and disadvantages of an antibody-based defense strategy (Table), and proposes stockpiling specific antibodies for use in the event of biological attacks.

**Table:** Therapeutic and prophylactic strategies to combat biological warfare agent.

Strategy	Advantages	Disadvantages
Antimicrobial chemotherapy	Relatively cheap Oral administration Self-administration	Potential for superinfection Selection for bystander resistance Continuous use needed for efficacy Drug-dependent side effects Organisms can be engineered for resistance Long development time to licensure
Vaccines	Long-lasting protection	Low public acceptance to theoretical threat? Immune response requires time Uncertain efficacy in compromised hosts Potential for deleterious immunologic sequelae Long development time to licensure
Passive antibody	Immediate protection, lasting weeks or months Low toxicity for human antibodies Versatility Shorter development time to licensure? Potential for self-administration	Will probably require cold chain Expensive Need for systemic administration Agents can be engineered for resistance Potential for infusion related reactions

## Activity of Specific Antibodies against Biological Warfare Agents

In the section below the evidence that humoral immunity is active against important biological agents is reviewed. Representative studies are cited for each pathogen.

### Anthrax

The three clinical forms of anthrax are cutaneous, gastrointestinal, and inhalational, caused by inoculation, ingestion, or inhalation of spores of Bacillus anthracis, respectively (reviewed in [5]). Anthrax virulence is determined by two toxins known as lethal factor (LF) and edema factor (EF). These toxins gain access to the cell through a third component known as protective antigen (PA), which binds to the cell surface receptor (6). Vaccination studies have established a direct correlation between antibody titer to PA and survival after lethal challenge with virulent anthrax spores (7,8). Passive administration of polyclonal antibodies raised against recombinant PA is protective in mice (9) and guinea pigs (10). Animals that received immune serum providing a titer >1:200 were fully protected. Immune serum containing antibodies to PA can be effective in the therapy of established experimental infection in guinea pigs when given as late as 24 h after intranasal spore inoculation (11). Evidence also indicates that some antibodies bind to anthrax spore proteins and prevent their germination, suggesting a role for antibody in interfering with the early stages of infection (12).

In contrast to the unequivocal results obtained with polyclonal sera in passive protection experiments, studies with MAbs have been somewhat disappointing. A recent study evaluated the protective efficacy of four murine MAbs to anthrax toxin components (two to PA and one each to EF and LF) in guinea pigs; only one (to PA) gave partial protection, and the effect was substantially lower than that observed with polyclonal sera (10). The relative lack of efficacy of MAbs to PA relative to the protection observed with polyclonal antibody preparations may reflect a need for antibody preparations with multiple neutralizing activities.

Overall, the results indicate that passive antibody can protect against anthrax. Serum therapy was used for the treatment of human anthrax with some success in the pre-antibiotic era in uncontrolled studies (13). The Centers for Disease Control and Prevention (CDC) has recently proposed generating antibody preparations for human therapeutic use from serum of persons vaccinated for anthrax (14). The most likely mechanism of action by which antibodies to anthrax toxin proteins mediate protection is binding to toxin and impeding its interaction with the host cell. However, the process of toxin-mediated damage has many possible steps when an antibody could interfere with the process. For example, an antibody to PA could prevent this protein from binding to its cellular receptor. This mechanism of action has been validated by experiments with single-chain antibody fragments containing the antibody binding site (15). However, the relative inefficacy of single MAbs suggests that highly active antibody preparations combining MAbs of different specificities may be necessary.

### Botulinum Toxins

These toxins are produced by Clostridium botulinum and encompass seven antigenic types known by the letters A through G (reviewed in [16]). The different toxins are defined by specific antisera that are not cross protective. Hence, antibody to toxin A does not neutralize the other toxins. Botulinum toxins are taken up by nerve cells through pinocytosis and mediate their action by binding to neuromuscular junctions and preventing acetylcholine release leading to muscular paralysis (16). The damage to the synaptic junction is considered to be irreversible, with recovery being the result of new axonal growth that may take weeks or months. Therapy for botulism is largely supportive, although prompt administration of an antitoxin may reduce the severity of symptoms by neutralizing unbound toxin in circulation. Antitoxin therapy for botulism lowers death rates and shortens the duration of symptoms when given within 24 h of the onset of disease (17). An equine trivalent antitoxin available from CDC contains neutralizing antibodies against the most common causes of human botulism, toxin types A, B, and E. For therapy of botulism caused by other toxin types, an experimental heptavalent equine serum is available (18). Given the side effects associated with the use of equine sera, there is great interest in the generation of human antibody preparations with neutralizing activity against the seven botulinum toxins (16). Passive administration of human botulinum immune globulin derived from volunteers vaccinated with pentavalent botulinum toxoid (ABCDE) vaccine has been protective in monkeys (19) and guinea pigs (20) against aerosolized botulinum toxin.

Many neutralizing MAbs to botulinum toxins have been generated that have potential diagnostic and therapeutic applications (21–24). The epitopes recognized by certain neutralizing antibodies have been mapped to conformational antigenic determinants (25). Recent reports indicate that biological activity of botulinum toxin can be enhanced by polyclonal equine antibody binding at equimolar concentrations of immunoglobulin (Ig) G and toxin protein (26). The proposed mechanism for this effect involves a conformational change upon antibody binding to certain epitopes, which translates into enhanced toxicity in vitro at low ratios of IgG to toxin protein. Although higher ratios of antibody to toxin produce neutralization in vitro and in vivo, this observation suggests the possibility that certain antibodies to botulinum toxin can be deleterious to the host and the need for adequate amounts in therapy. Interestingly, some MAbs can transiently reverse blockage of acetylcholine release when microinjected inside ganglionic neurons (21), raising the possibility that antibodies engineered for enhanced cellular penetration may have superior therapeutic properties.

### Brucellosis

Several species of *Brucella* can cause disease in humans, including *Brucella melitensis, B. suis, B. abortus,* and *B. canis.* Antibodies specific for the O polysaccharide of *B. abortus* are protective in mice (27). When administered before infection, MAbs to the M epitope of *Brucella* spp. reduce bacterial counts in the spleens of mice (28). A panel of murine MAbs to *B. melitensis* have been shown to be effective in protecting against experimental murine brucellosis (29). Other MAbs to a common epitope in *B. melitensis* and *B. abortus* have been shown to be protective (30). For the ram pathogen B. ovis, antibodies to rough lipopolysaccharide and to outer membrane proteins are protective in mice (31,32). These studies indicate the existence of multiple antigens in *Brucella* spp. that can elicit protective antibody responses.

### Q Fever

*Coxiella burnetii* is the causative agent of Q fever. Relatively little recent work has been conducted on the efficacy of specific antibody against *C. burnetii* infection. However, passive transfer of antibody protective against murine experimental infection with *C. burnetii* has been reported. Protection was observed in mice given agglutinating antibodies to Phase I C. burnetii (33). A second study extended those findings by demonstrating that passive antibody was effective in helping to clear murine infection only if given before or at the same time as a challenge with C. burnetii (34). Antibody-dependent cellular cytotoxicity of C. burnetii–infected macrophages suggests a potential mechanism by which humoral immunity can mediate protection (35). Notably, passive antibody was not effective in T cell–deficient mice, indicating that intact cellular immunity is needed for antibody function (34).

### Plague

Yersinia pestis is the causative agent of plague (reviewed in [36]). Horse serum was used for treating human plague in the pre-antibiotic era, particularly in India, where prompt administration of serum was reportedly associated with reduced mortality (37). In recent years, animal studies have conclusively established that certain antibodies are protective against Y. pestis. Protection against experimental Y. pestis infection in mice vaccinated with a subunit vaccine comprising the Fraction 1 and V antigens was shown to depend on the titer of serum IgG1 (38). Passive antibody administration protects severe combined immunodeficiency (SCID) mice against lethal Y. pestis infection (39). Importantly, passive antibody was protective against experimental pneumonic plague (39). In mice MAbs to Fraction 1 (F1) protein of Y. pestis were shown to protect against bubonic and pneumonic plague (40). Interestingly, F1– variants were recovered from some MAb-treated animals, suggesting that antibody could select for variants that lacked the epitope and thus illustrating a potential problem with therapy based on a single antibody.

### Smallpox

Variola is the causative agent of smallpox (reviewed in [41]). In the early 20th century, administration of convalescent-phase sera to patients with smallpox was claimed to shorten the course of the disease and abort the pustular stage (42). A recounting of anecdotal medical experience in Hong Kong by a British medical officer stated that serum administration was effective provided that the donor had had smallpox for at least 30 days (43). Another report from India describes a patient treated with both convalescent-phase sera and vaccinia immunization who reportedly recovered faster than expected (44). The experience with the use of vaccinia virus vaccine to prevent smallpox suggests that antibody preparations could be generated that would be active against variola virus. Vaccinia immune globulin from vaccinated volunteers has been used to treat vaccinia vaccination–associated disease (45). Most importantly, administration of vaccinia immune globulin to persons in close contact with smallpox patients substantially reduced the incidence of disease compared with rates in exposed persons who did not receive passive immunization (46). Neutralizing and protective antibodies to vaccinia virus have been described that target viral envelope antigens (47). The efficacy of specific antibody in aborting or modifying the course of vaccinia and variola infection provides a rationale for using passive antibody administration to prevent smallpox in conjunction with a vaccination strategy. This strategy is supported by the fact that immune globulin has an excellent record of preventing disease when used for postexposure prophylaxis against several viral diseases, including hepatitis and varicella zoster.

### Tularemia

*Francisella tularensis* is the causative agent of tularemia (48). Horse and goat immune sera were used for therapy of human tularemia as recently as the 1940s, with efficacy reported in selected patient groups (49). Passive administration of pooled murine immune sera protected mice against 10,000 50% lethal challenge doses (LD_50_ ) with the live vaccine strain (LVS) of *F. tularensis* (50). One antigen recognized by protective antibodies is bacterial lipopolysaccharide (50). The finding that antibodies to lipopolysaccharide protect against lethal challenge with LVS in mice has been confirmed, but the same antibodies are not protective against fully a virulent F. tularensis strain (51). Whether this finding reflects a limitation of the model used, insufficient amounts of specific antibody in immune sera, or efficacy of humoral immunity is not clear. Efficacy of passive antibody in protection against F. tularensis is dependent on cellular immunity, since no protection is observed in mice deficient in interferon gamma, CD4+, or CD8+ T cells (51,52). Despite the complexity of antibody action against *F. tularensis*, the observation that in certain circumstances passive antibody is protective suggests activity against this pathogen.

### Viral Encephalitides

Three viral meningoencephalitis syndromes are caused by alphaviruses: *Eastern equine encephalomyelitis virus* (EEEV), *Venezuelan equine encephalomyelitis virus* (VEEV), and *Western equine encephalomyelitis virus* (WEEV). Protective antibodies can be elicited by the alphaviruses that protect against lethal challenge in experimental murine models; one mechanism of action is interference with attachment (53,54). For EEEV, protection was associated with neutralizing and hemagglutination-inhibiting antibodies (53). For VEEV, protective antibodies have been shown to bind to a defined area of the E2 glycoprotein (55,56).

### Viral Hemorrhagic Fevers

Many viral agents are known to cause hemorrhagic fevers, including Ebola, Marburg, and Junin viruses. Passive antibody has been used for the treatment of Ebola (57), Argentine (58), and Lassa (59) hemorrhagic fevers, with encouraging results. Furthermore, considerable evidence from animal studies indicates that passive antibody administration prevents or ameliorates disease caused by viral agents of hemorrhagic fever (60–63). Studies in mice suggest that the protective efficacy of passive antibody action against Ebola virus (EBOV) is a result of suppression of viral growth that allows development of immunity (60). Hyperimmune goat serum generated by immunization with live EBOV protected guinea pigs against lethal challenge (64). Passive antibody therapy for EBOV infection may be effective in humans, as suggested by lower death rates in recipients of blood transfusions from convalescent patients (57). Two caveats in the use of passive antibody therapy with immune sera against hemorrhagic fevers that have emerged from studies in animal models are the existence of disease-enhancing antibodies (65) and the need for high-titer sera to achieve protection (66). However, problems with deleterious antibodies and insufficient activity could potentially be avoided by the use of MAb cocktails composed only of protective antibodies with high specific activity. In this regard, MAbs to EBOV have been developed that are protective in mice even when administered 2 days after infection (67).

### Biological Toxins

Toxin-binding antibodies have been known to be potent antitoxins since the landmark studies of Behring and Kitasato, which showed that immune sera protected against diphtheria (68). Antibody preparations continue to be used as antitoxins in the treatment of tetanus (69), diphtheria (69), botulism (18), and venomous bites (70). Specific antibodies remain the only therapeutic compounds available that are capable of neutralizing biological toxins in vivo. Hence, ample experience supports the notion that antibodies to biological toxins will protect against exposure to toxins produced by microbes used in biological warfare and may be useful for therapy of some toxin-mediated diseases.

A variety of toxins can be used for biological warfare, including ricin, trichothecene mycotoxins, and staphylococcal enterotoxins (71). MAbs to ricin have been described that protect mice against a lethal challenge with ricin toxin (72). Similarly, passive administration of MAbs to staphylococcal enterotoxin protects mice from lethal challenge with this toxin (73).

## Advantages of an Antibody-Based Defense Strategy

The above summary indicates that specific antibody can be effective against some of the major biological warfare agents. In fact, antibody preparations in the form of serum therapy were used historically for the treatment of anthrax (13), tularemia (49), and plague (37), albeit in uncontrolled trials that do not meet modern standards for establishing efficacy. The major advantage of passive antibody immunization in defense against biological weapons is that it provides a state of immediate immunity that can last for weeks and possibly months. Some human IgG isotypes have serum half-lives in excess of 30 days (74), which would confer long-lived protection to passively immunized persons. Antibodies are natural products with minimal toxicity, provided that they contain no aggregates and have no reactivity with host tissues. If vaccines are available, simultaneous administration of vaccine and antibody may be possible to provide both immediate and long-lasting protection, as is done for rabies in postexposure prophylaxis. Antibodies conjugated to enzymes, radionucleotides, or drugs could provide additional antimicrobial activities apart from those conferred by the native immunoglobulin molecule.

Although passive antibody will generally have to be given systemically, oral administration can be useful against certain gastrointestinal agents. With the exception of rabies antiserum, most antibody preparations in clinical use are given intravenously. The need for intravenous administration is a severe constraint for mass passive immunization and would likely limit this practice to a few recipients. However, this disadvantage may potentially be circumvented because Ig preparations can theoretically administered intramuscularly. Hence, generating antibody preparations suitable for delivery into one of the large muscles of the arm or leg may be possible without the need for logistically complicated intravenous infusions. Such antibody preparations could be supplied in self-injectable devices that could allow susceptible persons to protect themselves upon notification of a biological attack. However, for this scenario to be realistic, antibody preparations with high specific activity would have to be developed that would allow administration in a small volume.

An antibody-based defense strategy against biological warfare agents can be supported by a mature technology. Antibody-based therapies were first used in the late 19th century, and more than 100 years of experience has been gained in the development of therapeutic antibodies. In the past, antibody-based therapies were dependent on immune serum that was limited in availability and was associated with substantial side effects when the serum originated from animals (2,3). In recent years, major technical advances in the ability to generate antibodies include the development of a variety of expression systems, including hybridoma, bacteria, and phage systems (75,76). Since 1997, eight MAbs have been licensed for human therapeutic use; three of these are mouse-human chimerics and five are humanized murine MAbs (4). Each of these molecules has been the product of advances in biotechnology, and their success supports the view that the technology is in place for implementing an antibody-based defense strategy.

Immunoglobulins are highly versatile effector molecules that can be adapted for use against virtually any infectious agent or toxin. In fact, antibody therapy is now available for a variety of situations in which natural antibody immunity is not likely to be effective, including prevention of re-stenosis after coronary angioplasty and the therapy for venomous animal bites, digitalis toxicity, breast cancer, and Crohn disease (reviewed in [77]). Furthermore, the fact that natural protection to a given pathogen may rely on cell-mediated immunity does not negate the fact that passive antibody can still mediate protection. For example, protective MAbs have now been identified against such intracellular pathogens as Ehrlichia chaffeensis (78), Cryptococcus neoformans (79), Listeria monocytogenes (80), Candida albicans (81), and Mycobacterium tuberculosis (82), for which cell-mediated immunity is critically important for protection.

## Barriers to Developing an Antibody-Based Defensive Strategy

The use of antibody-based therapies against infectious agents in routine clinical practice is limited by several factors, including cost, need for a specific diagnosis before use, and the fact that passive immunization is more effective as prophylaxis than as therapy for established infections. Furthermore, availability of cheap antimicrobial chemotherapy for many common pathogens has reduced interest in developing antibody therapies against infectious diseases. In fact, of the 10 MAbs currently licensed for human use in the United States, only one is for an infectious disease (prophylaxis of respiratory syncytial virus infections) (4). However, these disadvantages do not necessarily apply in facing biological warfare or bioterrorism. Therapeutic immunoglobulins are undoubtedly among the most expensive drugs used in clinical practice. The high expense of Ig preparations is related to the fact that these reagents are more fragile than small molecular weight compounds and that they originate from immune donors or cell culture production and hence are costly to obtain, produce, and maintain. In addition, many of the indications for which immunoglobulins are used represent relatively small markets, and the cost efficiency associated with mass production may not apply.

One difficulty that has plagued the development of antibody-based therapies in infectious diseases is that the market size for an antibody reagent is proportional to the prevalence of disease (3). Since antibody reagents are almost always pathogen specific, the market for antibody-based therapies is often much smaller than that for drugs with broad antimicrobial activity. Small market size combined with high price and the availability of many antimicrobial drugs has not encouraged development of antibody-based therapies for many infectious diseases. However, in considering antibodies for biological defense, the market size equals the potentially vulnerable population. This consideration, combined with the fact that stockpiles would have to be replenished periodically as a result of lot expirations, could make the economic outlook more attractive to industry. Production of sufficient antibody protein for universal protection of the U.S. population against a specific biological agent would involve large-scale production and could result in cheaper unit prices.

Another problem associated with the high specificity of antibodies is that the agent would have to be identified before antibody use. However, awareness of an attack implies that the biological agent is likely to be detected once the first exposed persons become ill and a diagnosis is made. Furthermore, the number of agents likely to be employed in biological warfare or terrorism is relatively small, and it may be possible to deduce the identity of the agent rapidly. If the threat involves more than one agent, it is theoretically possible to design cocktails of immunoglobulins to protect against the likely culprits.

One aspect that has limited enthusiasm for antibody-based therapies against infectious agents is the recognition that the efficacy of an antibody is largely a function of timing of administration relative to the development of clinical symptoms. In this regard, immune sera was effective against pneumococcal pneumonia only when administered in the first 3 days after the onset of symptoms (reviewed [2,3]). For Shiga toxin-producing strains of Escherichia coli, the efficacy of passive antibody is largely a function of the time of administration and the dose given, with antibody efficacy declining rapidly when administered after the second day of infection (83). In fact, antibody to toxins may not be effective therapeutically once the toxin has bound to its receptor, as is the case for botulism, a condition for which late antibody therapy is relatively ineffective. However, in the event of a biological attack, the many exposed persons could likely be given antibody before the onset of symptoms. Despite reduced efficacy when administered after the onset of symptoms, antibody-based therapy is still useful for certain diseases, as evidenced by the fact that specific immunoglobulins are used for treatment of botulism (17,18), tetanus (84), Ebola hemorrhagic fever (57), and parvovirus-associated anemia in patients with AIDS (85,86).

The availability of antimicrobial therapy does not diminish the advantages of antibody-based therapies. Currently no drugs are available that specifically counteract the activity of preformed toxins, while toxin neutralization is a classical property of antibody-mediated immunity. For certain conditions, antibody therapy may have some advantages over antimicrobial therapy. For example, administration of human IgG may require only a one-time infusion, whereas antimicrobial therapy is likely to require continuous administration during the period of exposure and following infection. Furthermore, bacteria can be relatively easily engineered for resistance to antibiotic drugs. These issues were highlighted during the recent anthrax exposures, when 60 days of therapy was recommended after exposure, with a drug (e.g., ciprofloxacin) that was selected because of concerns about potential resistance in certain strains of B. anthracis (87). Prolonged use of antimicrobial drugs for prophylaxis against biological warfare agents such as anthrax carries inherent risks of drug toxicity and selection for drug-resistant strains among the host microbial flora (87).

Antibody defense strategies can be circumvented by the generation of agents that exhibit antigenic variation. MAbs that recognize a critical domain in a microbial antigen are particularly vulnerable to the emergence of antigenic variation arising from selection during person-to-person spread or genetic engineering of the biological agent. Hence, stockpiles of MAbs can easily be made obsolete by biological agents that exhibit antigenic differences. This problem may be circumvented by using polyclonal antibody preparations or MAb cocktails that bind multiple epitopes in the targeted antigen. The efficacy of antibody preparations can be safeguarded by classifying the binding specificities and characteristics of antibody preparations as state secrets. Furthermore, the possibility of counterstrategies should be incorporated into the design of antibody therapeutics by specifically targeting constant epitopes that are unlikely to retain biological activity if altered. In fact, it may be possible to safeguard the usefulness of antibody preparations designed specifically for protection against biological agents by concealing their specificity in complex preparations that defy immunologic analysis.

Currently, we lack sufficient immunologic knowledge to predict the specificities and isotypes that are protective against individual pathogens. Hence, the search for protective antibodies remains empirical. Incidentally, the identification of a protective antibody de facto identifies an antigen that is capable of eliciting a protective antibody response. In the case of C. neoformans and C. albicans, MAbs to polysaccharide components were first shown to be protective and this information was used to generate conjugate vaccine that were protective in mice (88,89). Hence, a search for therapeutic MAbs can lead to an useful reagent for immediate use and also identify antigens suitable for vaccine development.

Perhaps the greatest hurdle facing the development of antibody therapies, vaccines, and new antimicrobial therapies for many agents of biological warfare is that these compounds would have to be developed without standard clinical trials. Extrapolating from observations made in animal models and in vitro is treacherous because we do not understand the correlates of protection for the overwhelming majority of infectious agents. Our state of immunologic knowledge is not sufficiently advanced to predict which antibodies or vaccines would be effective in humans. However, efficacy in animals and in vitro does mean potential efficacy in humans. Hence, in the event of an emergency it is probably better to have compounds that are effective in animal models than to have no therapies at all. In the pre-antibiotic era, the mouse pneumococcal model accurately predicted the efficacy of horse serum in humans, and the dosing of horse antipneumococcal serum was based on units derived from the mouse protection test (2).

## Polyclonal versus MAb Products

In common usage, the term polyclonal antibody preparation refers to immune sera that usually contain pathogen-specific antibodies of various isotypes and specificities. In contrast, MAb preparations consist of a single immunoglobulin type, representing one isotype with one specificity. In theory, polyclonal preparations for human therapeutic use can be generated by mixing MAbs. Each product has important advantages and disadvantages that must be weighed in considering the development of a passive antibody strategy.

Polyclonal preparations have the advantage of consisting of diverse immunoglobulins that target different antigens; the heterogeneity in isotype composition confers broader biological activity through the various constant regions. Polyclonal preparations are generally relatively easy to make, provided that immune donors are available. However, the amount of specific antibodies in a polyclonal preparation usually represents only a minute fraction of the total antibody protein. Hence, polyclonal preparations tend to have low specific activity relative to MAb preparations. For example, in a comparison of the activity of human MAbs with that of human immune globulin, 0.7 mg of a mixture of two MAbs had the same neutralizing activity as 100 mg–170 mg of tetanus immune globulin (90). Other problems associated with polyclonal preparations generated from immune donors are lot-to-lot variations in the amount of specific antibody (91), limited supply (92), and the possibility of transmission of infectious agents (93).

MAbs have the advantage that they can be defined precisely with regard to structure, specificity and activity. Furthermore, MAbs produced in vitro by hybridomas or other expression systems can provide an inexhaustible supply of immunoglobulin, thus freeing production from relying on a limited number of immune donors. However, the fact that MAbs recognize only a single epitope means that they have limited usefulness against pathogens that exhibit antigenic variation. This problem can be circumvented by generating MAb cocktails, with the caveat that such preparations would likely encounter a more complex regulatory process.

## Proposal for an Antibody-Based Defensive Strategy

Stockpiling antibody-based reagents that can be rapidly administered to exposed populations would substantially reduce the threat of many biological agents by providing a means of conferring immediate immunity to susceptible persons. For persistent threats for which vaccines are available, this measure would provide additional time for immunization, as well as reducing the threat. Development of antibody-based therapies may reduce the attractiveness of biological warfare as a weapon of terror by providing antidotes to help neutralize the threat. An aggressor could attempt to defeat a passive antibody defense by engineering the agent to express antigenic changes, proteases, or antibody-binding proteins. However, in this arms race the advantage may favor the defender, since it is technologically easier to generate a new antibody effective against the changed agent than to engineer a pathogen or agent to enhance virulence. Antigenic changes by definition create new epitopes that can be targeted by other antibodies. Antibodies can also be engineered to resist proteolysis by altering the amino acid sequence to eliminate proteolytic sites. In fact, a neutralizing antibody preparation can likely be generated much faster than new biological agents can be developed. An example of the rapidity with which therapeutic antibodies can be developed comes from the 1905 epidemic of meningococcal meningitis in New York City, when Flexner generated an effective horse antiserum within months and used it to treat patients before the epidemic abated naturally (94). Although this example is not applicable today, given regulations on the development of therapeutics, it provides a dramatic example of the concept that antibody therapies can be developed quickly. The development of antibody-based therapies relies on technology that can respond rapidly to new threats, whereas construction of new biological agents would almost certainly require considerable basic research and development. The same may not apply to new antimicrobial chemotherapy or vaccines, which often require substantially longer development times.
